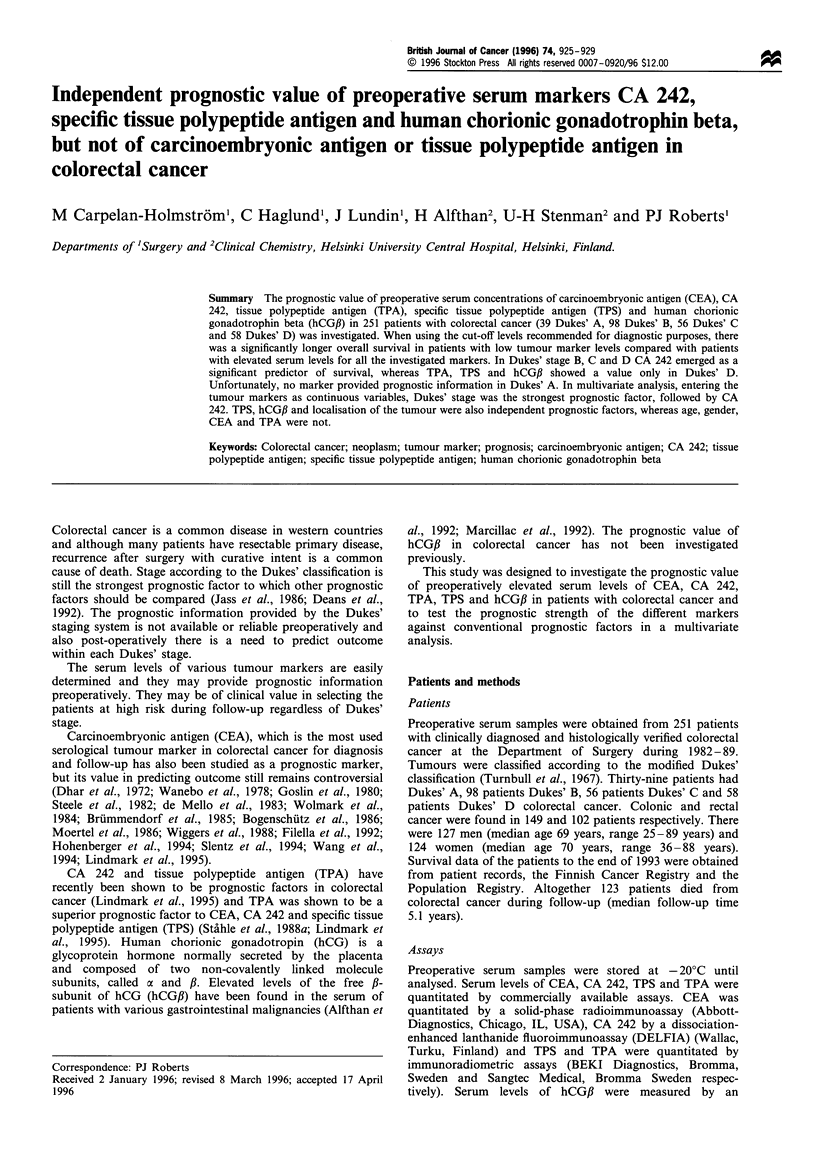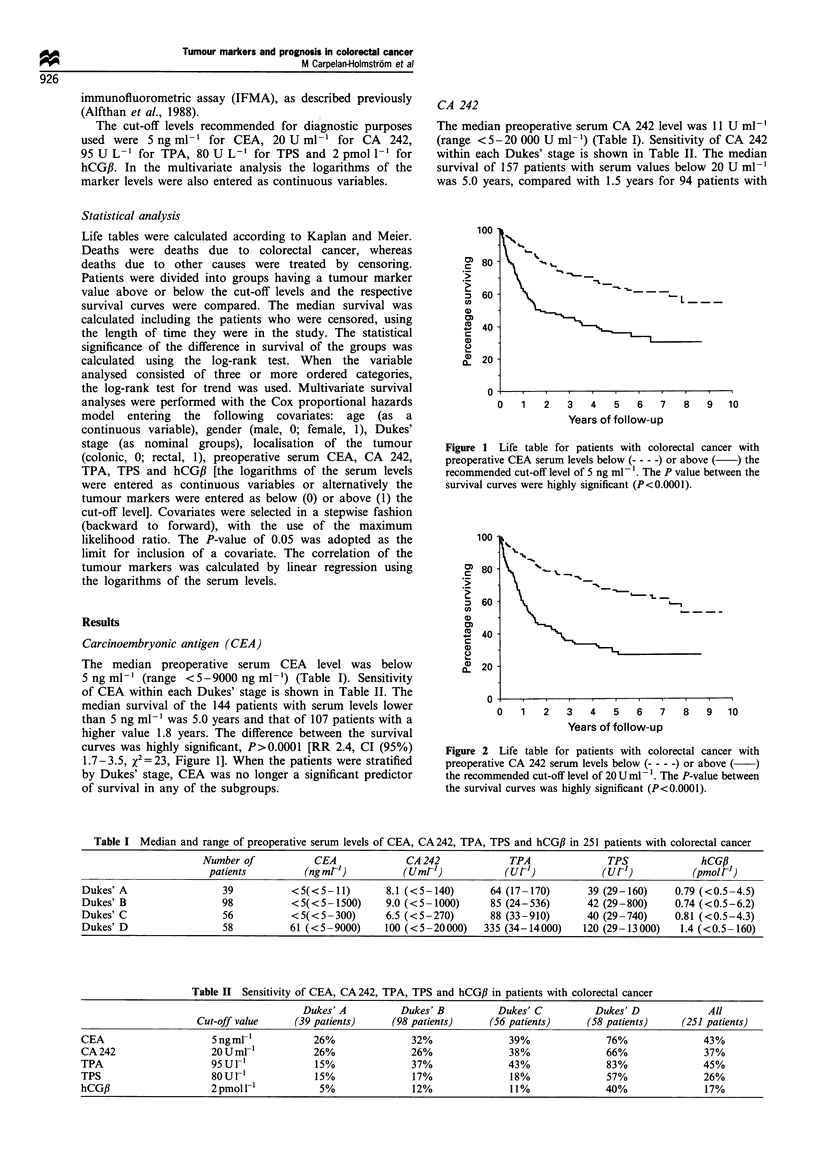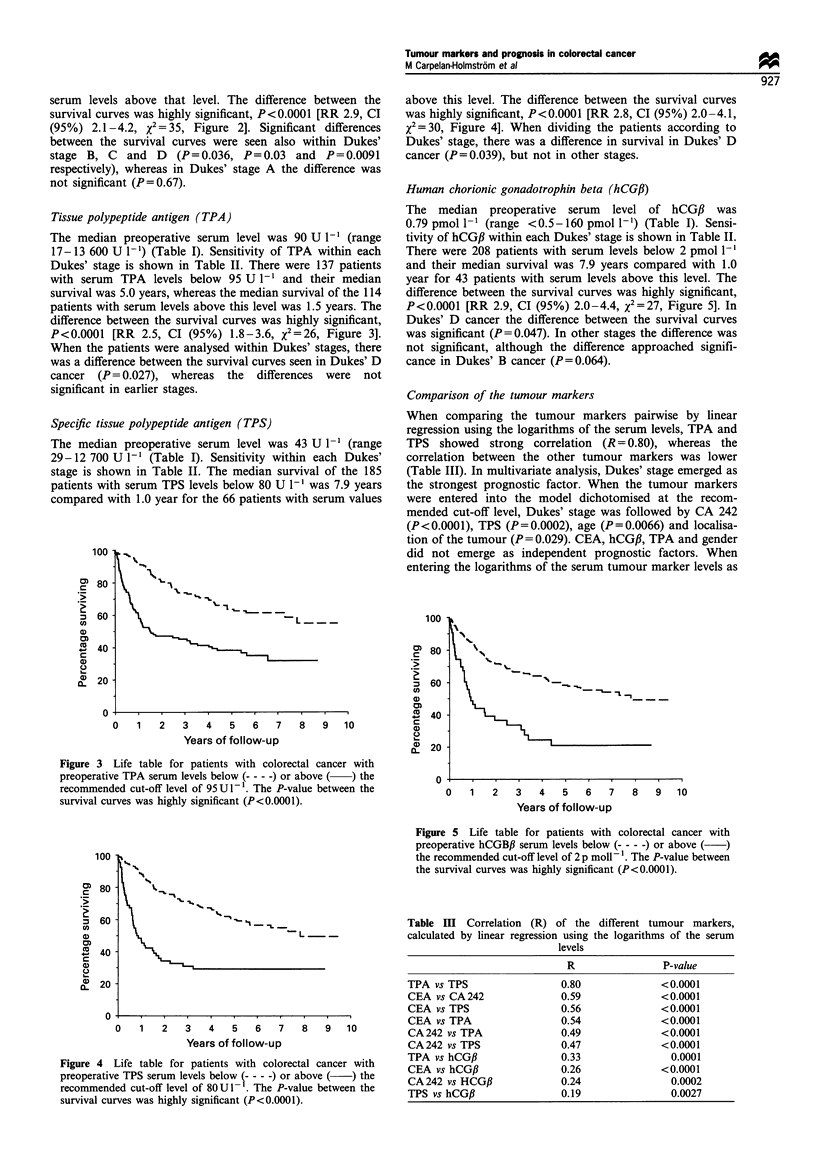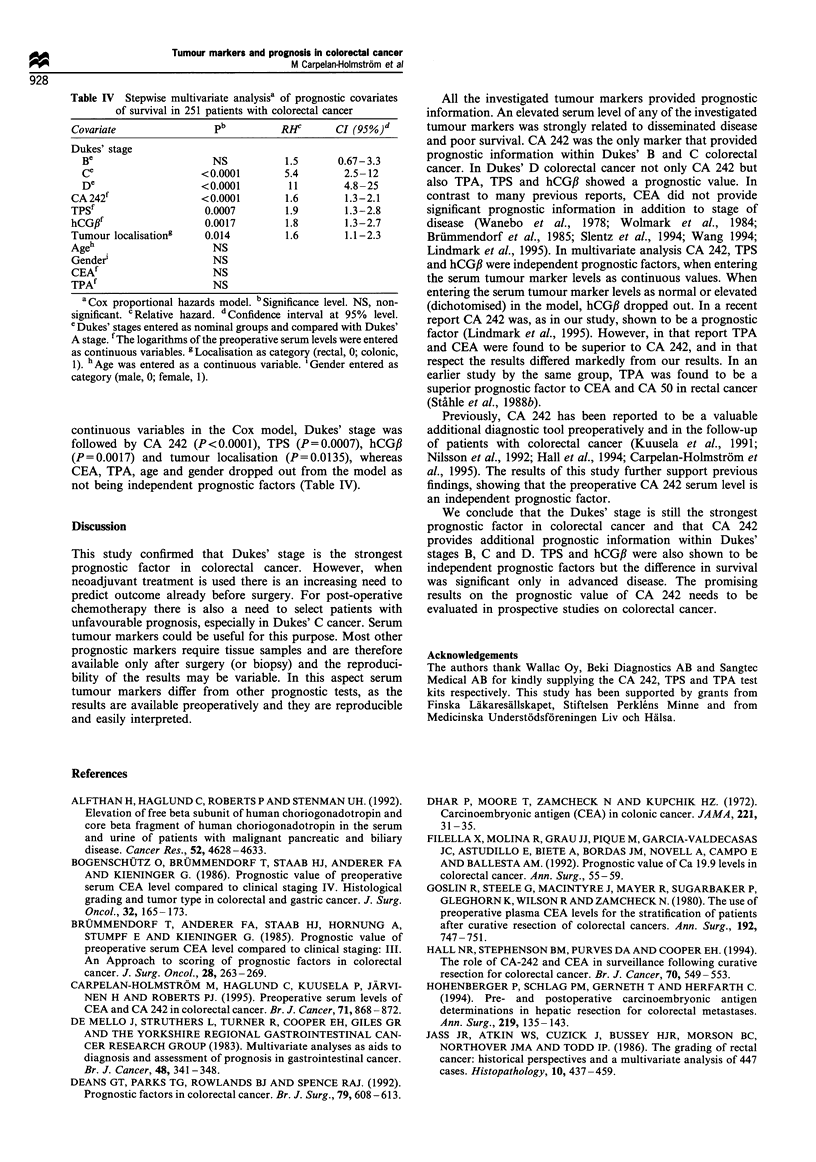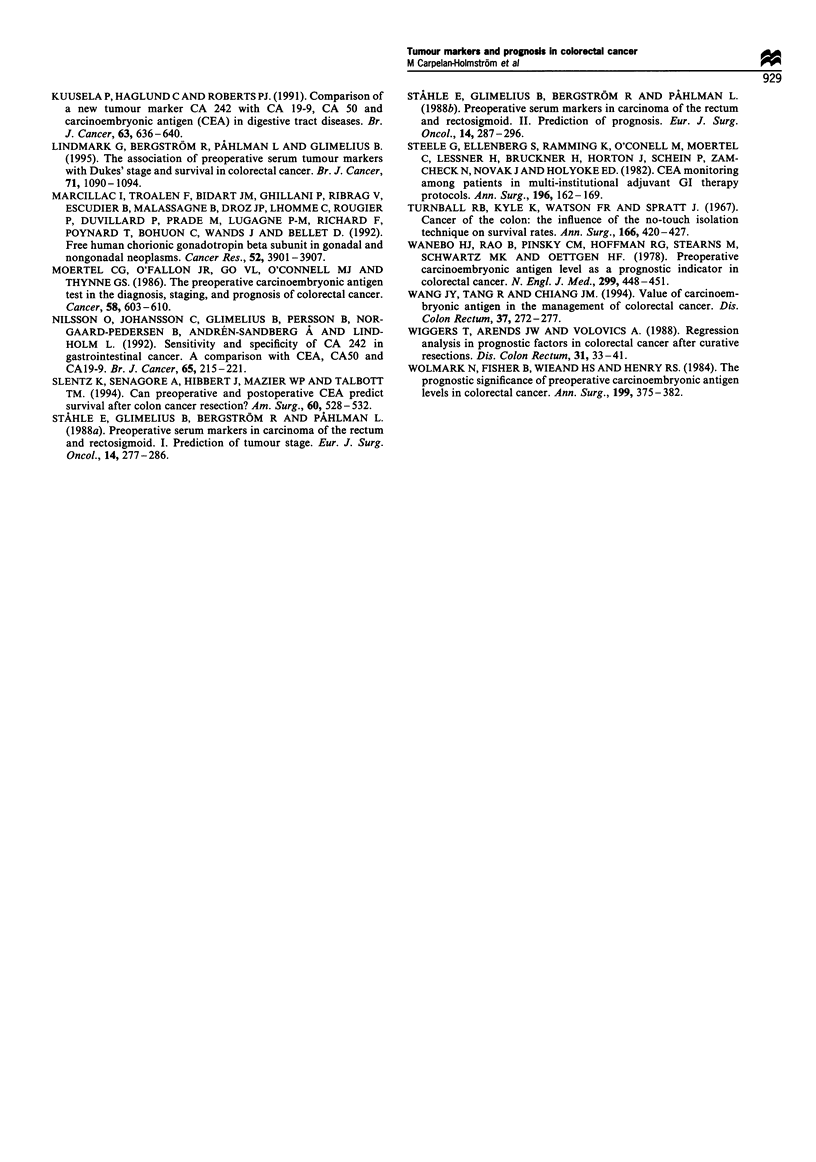# Independent prognostic value of preoperative serum markers CA 242, specific tissue polypeptide antigen and human chorionic gonadotrophin beta, but not of carcinoembryonic antigen or tissue polypeptide antigen in colorectal cancer.

**DOI:** 10.1038/bjc.1996.458

**Published:** 1996-09

**Authors:** M. Carpelan-Holmström, C. Haglund, J. Lundin, H. Alfthan, U. H. Stenman, P. J. Roberts

**Affiliations:** Department of Surgery, Helsinki University Central Hospital, Finland.

## Abstract

The prognostic value of preoperative serum concentrations of carcinoembryonic antigen (CEA), CA 242, tissue polypeptide antigen (TPA), specific tissue polypeptide antigen (TPS) and human chorionic gonadotrophin beta (hCG beta) in 251 patients with colorectal cancer (39 Dukes' A, 98 Dukes' B, 56 Dukes' C and 58 Dukes' D) was investigated. When using the cut-off levels recommended for diagnostic purposes, there was a significantly longer overall survival in patients with low tumour marker levels compared with patients with elevated serum levels for all the investigated markers. In Dukes' stage B, C and D CA 242 emerged as a significant predictor of survival, whereas TPA, TPS and hCG beta showed a value only in Dukes' D. Unfortunately, no marker provided prognostic information in Dukes' A. In multivariate analysis, entering the tumour markers as continuous variables, Dukes' stage was the strongest prognostic factor, followed by CA 242. TPS, hCG beta and localisation of the tumour were also independent prognostic factors, whereas age, gender, CEA and TPA were not.


British Journal of Cancer (1996) 74, 925-929
© 1996 Stockton Press All rights reserved 0007-0920/96 $12.00

### Independent prognostic value of preoperative serum markers CA 242, specific tissue polypeptide antigen and human chorionic gonadotrophin beta, but not of carcinoembryonic antigen or tissue polypeptide antigen in colorectal cancer

M Carpelan-Holmström[1], C Haglund[1], J Lundin[1], H Alfthan[2], U-H Stenman[2] and PJ Roberts[1]

*Departments of [1]Surgery and [2]Clinical Chemistry, Helsinki University Central Hospital, Helsinki, Finland.*

**Summary** The prognostic value of preoperative serum concentrations of carcinoembryonic antigen (CEA), CA 242, tissue polypeptide antigen (TPA), specific tissue polypeptide antigen (TPS) and human chorionic gonadotrophin beta (hCG$\beta$) in 251 patients with colorectal cancer (39 Dukes' A, 98 Dukes' B, 56 Dukes' C and 58 Dukes' D) was investigated. When using the cut-off levels recommended for diagnostic purposes, there was a significantly longer overall survival in patients with low tumour marker levels compared with patients with elevated serum levels for all the investigated markers. In Dukes' stage B, C and D CA 242 emerged as a significant predictor of survival, whereas TPA, TPS and hCG$\beta$ showed a value only in Dukes' D. Unfortunately, no marker provided prognostic information in Dukes' A. In multivariate analysis, entering the tumour markers as continuous variables, Dukes' stage was the strongest prognostic factor, followed by CA 242. TPS, hCG$\beta$ and localisation of the tumour were also independent prognostic factors, whereas age, gender, CEA and TPA were not.

**Keywords:** Colorectal cancer; neoplasm; tumour marker; prognosis; carcinoembryonic antigen; CA 242; tissue polypeptide antigen; specific tissue polypeptide antigen; human chorionic gonadotrophin beta

Colorectal cancer is a common disease in western countries and although many patients have resectable primary disease, recurrence after surgery with curative intent is a common cause of death. Stage according to the Dukes' classification is still the strongest prognostic factor to which other prognostic factors should be compared (Jass *et al.*, 1986; Deans *et al.*, 1992). The prognostic information provided by the Dukes' staging system is not available or reliable preoperatively and also post-operatively there is a need to predict outcome within each Dukes' stage.

The serum levels of various tumour markers are easily determined and they may provide prognostic information preoperatively. They may be of clinical value in selecting the patients at high risk during follow-up regardless of Dukes' stage.

Carcinoembryonic antigen (CEA), which is the most used serological tumour marker in colorectal cancer for diagnosis and follow-up has also been studied as a prognostic marker, but its value in predicting outcome still remains controversial (Dhar *et al.*, 1972; Wanebo *et al.*, 1978; Goslin *et al.*, 1980; Steele *et al.*, 1982; de Mello *et al.*, 1983; Wolmark *et al.*, 1984; Brümmendorf *et al.*, 1985; Bogenschütz *et al.*, 1986; Moertel *et al.*, 1986; Wiggers *et al.*, 1988; Filella *et al.*, 1992; Hohenberger *et al.*, 1994; Slentz *et al.*, 1994; Wang *et al.*, 1994; Lindmark *et al.*, 1995).

CA 242 and tissue polypeptide antigen (TPA) have recently been shown to be prognostic factors in colorectal cancer (Lindmark *et al.*, 1995) and TPA was shown to be a superior prognostic factor to CEA, CA 242 and specific tissue polypeptide antigen (TPS) (Ståhle *et al.*, 1988*a*; Lindmark *et al.*, 1995). Human chorionic gonadotropin (hCG) is a glycoprotein hormone normally secreted by the placenta and composed of two non-covalently linked molecule subunits, called $\alpha$ and $\beta$. Elevated levels of the free $\beta$-subunit of hCG (hCG$\beta$) have been found in the serum of patients with various gastrointestinal malignancies (Alfthan *et al.*, 1992; Marcillac *et al.*, 1992). The prognostic value of hCG$\beta$ in colorectal cancer has not been investigated previously.

This study was designed to investigate the prognostic value of preoperatively elevated serum levels of CEA, CA 242, TPA, TPS and hCG$\beta$ in patients with colorectal cancer and to test the prognostic strength of the different markers against conventional prognostic factors in a multivariate analysis.

### Patients and methods

### *Patients*

Preoperative serum samples were obtained from 251 patients with clinically diagnosed and histologically verified colorectal cancer at the Department of Surgery during 1982-89. Tumours were classified according to the modified Dukes' classification (Turnbull *et al.*, 1967). Thirty-nine patients had Dukes' A, 98 patients Dukes' B, 56 patients Dukes' C and 58 patients Dukes' D colorectal cancer. Colonic and rectal cancer were found in 149 and 102 patients respectively. There were 127 men (median age 69 years, range 25-89 years) and 124 women (median age 70 years, range 36-88 years). Survival data of the patients to the end of 1993 were obtained from patient records, the Finnish Cancer Registry and the Population Registry. Altogether 123 patients died from colorectal cancer during follow-up (median follow-up time 5.1 years).

### *Assays*

Preoperative serum samples were stored at −20°C until analysed. Serum levels of CEA, CA 242, TPS and TPA were quantitated by commercially available assays. CEA was quantitated by a solid-phase radioimmunoassay (Abbott-Diagnostics, Chicago, IL, USA), CA 242 by a dissociation-enhanced lanthanide fluoroimmunoassay (DELFIA) (Wallac, Turku, Finland) and TPS and TPA were quantitated by immunoradiometric assays (BEKI Diagnostics, Bromma, Sweden and Sangtec Medical, Bromma Sweden respectively). Serum levels of hCG$\beta$ were measured by an

Correspondence: PJ Roberts
Received 2 January 1996; revised 8 March 1996; accepted 17 April 1996

immunofluorometric assay (IFMA), as described previously (Alfthan et al., 1988).

The cut-off levels recommended for diagnostic purposes used were 5 ng ml$^{-1}$ for CEA, 20 U ml$^{-1}$ for CA 242, 95 U L$^{-1}$ for TPA, 80 U L$^{-1}$ for TPS and 2 pmol l$^{-1}$ for hCG$\beta$. In the multivariate analysis the logarithms of the marker levels were also entered as continuous variables.

*Statistical analysis*

Life tables were calculated according to Kaplan and Meier. Deaths were deaths due to colorectal cancer, whereas deaths due to other causes were treated by censoring. Patients were divided into groups having a tumour marker value above or below the cut-off levels and the respective survival curves were compared. The median survival was calculated including the patients who were censored, using the length of time they were in the study. The statistical significance of the difference in survival of the groups was calculated using the log-rank test. When the variable analysed consisted of three or more ordered categories, the log-rank test for trend was used. Multivariate survival analyses were performed with the Cox proportional hazards model entering the following covariates: age (as a continuous variable), gender (male, 0; female, 1), Dukes' stage (as nominal groups), localisation of the tumour (colonic, 0; rectal, 1), preoperative serum CEA, CA 242, TPA, TPS and hCG$\beta$ [the logarithms of the serum levels were entered as continuous variables or alternatively the tumour markers were entered as below (0) or above (1) the cut-off level]. Covariates were selected in a stepwise fashion (backward to forward), with the use of the maximum likelihood ratio. The $P$-value of 0.05 was adopted as the limit for inclusion of a covariate. The correlation of the tumour markers was calculated by linear regression using the logarithms of the serum levels.

### Results

*Carcinoembryonic antigen (CEA)*

The median preoperative serum CEA level was below 5 ng ml$^{-1}$ (range <5–9000 ng ml$^{-1}$) (Table I). Sensitivity of CEA within each Dukes' stage is shown in Table II. The median survival of the 144 patients with serum levels lower than 5 ng ml$^{-1}$ was 5.0 years and that of 107 patients with a higher value 1.8 years. The difference between the survival curves was highly significant, $P$>0.0001 [RR 2.4, CI (95%) 1.7–3.5, $\chi^2$=23, Figure 1]. When the patients were stratified by Dukes' stage, CEA was no longer a significant predictor of survival in any of the subgroups.

*CA 242*

The median preoperative serum CA 242 level was 11 U ml$^{-1}$ (range <5–20 000 U ml$^{-1}$) (Table I). Sensitivity of CA 242 within each Dukes' stage is shown in Table II. The median survival of 157 patients with serum values below 20 U ml$^{-1}$ was 5.0 years, compared with 1.5 years for 94 patients with

**Figure 1** Life table for patients with colorectal cancer with preoperative CEA serum levels below (- - - -) or above (——) the recommended cut-off level of 5 ng ml$^{-1}$. The $P$ value between the survival curves were highly significant ($P$<0.0001).

**Figure 2** Life table for patients with colorectal cancer with preoperative CA 242 serum levels below (- - - -) or above (——) the recommended cut-off level of 20 U ml$^{-1}$. The $P$-value between the survival curves was highly significant ($P$<0.0001).

**Table I** Median and range of preoperative serum levels of CEA, CA 242, TPA, TPS and hCG$\beta$ in 251 patients with colorectal cancer

| | *Number of patients* | *CEA (ng ml$^{-1}$)* | *CA 242 (U ml$^{-1}$)* | *TPA (U l$^{-1}$)* | *TPS (U l$^{-1}$)* | *hCG$\beta$ (pmol l$^{-1}$)* |
|---|---|---|---|---|---|---|
| Dukes' A | 39 | <5(<5–11) | 8.1 (<5–140) | 64 (17–170) | 39 (29–160) | 0.79 (<0.5–4.5) |
| Dukes' B | 98 | <5(<5–1500) | 9.0 (<5–1000) | 85 (24–536) | 42 (29–800) | 0.74 (<0.5–6.2) |
| Dukes' C | 56 | <5(<5–300) | 6.5 (<5–270) | 88 (33–910) | 40 (29–740) | 0.81 (<0.5–4.3) |
| Dukes' D | 58 | 61 (<5–9000) | 100 (<5–20000) | 335 (34–14000) | 120 (29–13000) | 1.4 (<0.5–160) |

**Table II** Sensitivity of CEA, CA 242, TPA, TPS and hCG$\beta$ in patients with colorectal cancer

| | *Cut-off value* | *Dukes' A (39 patients)* | *Dukes' B (98 patients)* | *Dukes' C (56 patients)* | *Dukes' D (58 patients)* | *All (251 patients)* |
|---|---|---|---|---|---|---|
| CEA | 5 ng ml$^{-1}$ | 26% | 32% | 39% | 76% | 43% |
| CA 242 | 20 U ml$^{-1}$ | 26% | 26% | 38% | 66% | 37% |
| TPA | 95 U l$^{-1}$ | 15% | 37% | 43% | 83% | 45% |
| TPS | 80 U l$^{-1}$ | 15% | 17% | 18% | 57% | 26% |
| hCG$\beta$ | 2 pmol l$^{-1}$ | 5% | 12% | 11% | 40% | 17% |

serum levels above that level. The difference between the survival curves was highly significant, $P<0.0001$ [RR 2.9, CI (95%) 2.1–4.2, $\chi^2=35$, Figure 2]. Significant differences between the survival curves were seen also within Dukes' stage B, C and D ($P=0.036$, $P=0.03$ and $P=0.0091$ respectively), whereas in Dukes' stage A the difference was not significant ($P=0.67$).

*Tissue polypeptide antigen (TPA)*

The median preoperative serum level was 90 U $l^{-1}$ (range 17–13 600 U $l^{-1}$) (Table I). Sensitivity of TPA within each Dukes' stage is shown in Table II. There were 137 patients with serum TPA levels below 95 U $l^{-1}$ and their median survival was 5.0 years, whereas the median survival of the 114 patients with serum levels above this level was 1.5 years. The difference between the survival curves was highly significant, $P<0.0001$ [RR 2.5, CI (95%) 1.8–3.6, $\chi^2=26$, Figure 3]. When the patients were analysed within Dukes' stages, there was a difference between the survival curves seen in Dukes' D cancer ($P=0.027$), whereas the differences were not significant in earlier stages.

*Specific tissue polypeptide antigen (TPS)*

The median preoperative serum level was 43 U $l^{-1}$ (range 29–12 700 U $l^{-1}$ (Table I). Sensitivity within each Dukes' stage is shown in Table II. The median survival of the 185 patients with serum TPS levels below 80 U $l^{-1}$ was 7.9 years compared with 1.0 year for the 66 patients with serum values above this level. The difference between the survival curves was highly significant, $P<0.0001$ [RR 2.8, CI (95%) 2.0–4.1, $\chi^2=30$, Figure 4]. When dividing the patients according to Dukes' stage, there was a difference in survival in Dukes' D cancer ($P=0.039$), but not in other stages.

*Human chorionic gonadotrophin beta (hCGβ)*

The median preoperative serum level of hCGβ was 0.79 pmol $l^{-1}$ (range <0.5–160 pmol $l^{-1}$) (Table I). Sensitivity of hCGβ within each Dukes' stage is shown in Table II. There were 208 patients with serum levels below 2 pmol $l^{-1}$ and their median survival was 7.9 years compared with 1.0 year for 43 patients with serum levels above this level. The difference between the survival curves was highly significant, $P<0.0001$ [RR 2.9, CI (95%) 2.0–4.4, $\chi^2=27$, Figure 5]. In Dukes' D cancer the difference between the survival curves was significant ($P=0.047$). In other stages the difference was not significant, although the difference approached significance in Dukes' B cancer ($P=0.064$).

*Comparison of the tumour markers*

When comparing the tumour markers pairwise by linear regression using the logarithms of the serum levels, TPA and TPS showed strong correlation ($R=0.80$), whereas the correlation between the other tumour markers was lower (Table III). In multivariate analysis, Dukes' stage emerged as the strongest prognostic factor. When the tumour markers were entered into the model dichotomised at the recommended cut-off level, Dukes' stage was followed by CA 242 ($P<0.0001$), TPS ($P=0.0002$), age ($P=0.0066$) and localisation of the tumour ($P=0.029$). CEA, hCGβ, TPA and gender did not emerge as independent prognostic factors. When entering the logarithms of the serum tumour marker levels as

**Figure 3** Life table for patients with colorectal cancer with preoperative TPA serum levels below (- - - -) or above (——) the recommended cut-off level of 95 U $l^{-1}$. The *P*-value between the survival curves was highly significant ($P<0.0001$).

**Figure 4** Life table for patients with colorectal cancer with preoperative TPS serum levels below (- - - -) or above (——) the recommended cut-off level of 80 U $l^{-1}$. The *P*-value between the survival curves was highly significant ($P<0.0001$).

**Figure 5** Life table for patients with colorectal cancer with preoperative hCGBβ serum levels below (- - - -) or above (——) the recommended cut-off level of 2 p moll$^{-1}$. The *P*-value between the survival curves was highly significant ($P<0.0001$).

**Table III** Correlation (R) of the different tumour markers, calculated by linear regression using the logarithms of the serum levels

| | R | P-*value* |
|---|---|---|
| TPA *vs* TPS | 0.80 | <0.0001 |
| CEA *vs* CA 242 | 0.59 | <0.0001 |
| CEA *vs* TPS | 0.56 | <0.0001 |
| CEA *vs* TPA | 0.54 | <0.0001 |
| CA 242 *vs* TPA | 0.49 | <0.0001 |
| CA 242 *vs* TPS | 0.47 | <0.0001 |
| TPA *vs* hCGβ | 0.33 | 0.0001 |
| CEA *vs* hCGβ | 0.26 | <0.0001 |
| CA 242 *vs* HCGβ | 0.24 | 0.0002 |
| TPS *vs* hCGβ | 0.19 | 0.0027 |

Table IV Stepwise multivariate analysis[a] of prognostic covariates of survival in 251 patients with colorectal cancer

| *Covariate* | P[b] | *RH*[c] | *CI (95%)*[d] |
|---|---|---|---|
| Dukes' stage | | | |
| B[e] | NS | 1.5 | 0.67–3.3 |
| C[e] | <0.0001 | 5.4 | 2.5–12 |
| D[e] | <0.0001 | 11 | 4.8–25 |
| CA 242[f] | <0.0001 | 1.6 | 1.3–2.1 |
| TPS[f] | 0.0007 | 1.9 | 1.3–2.8 |
| hCGβ[f] | 0.0017 | 1.8 | 1.3–2.7 |
| Tumour localisation[g] | 0.014 | 1.6 | 1.1–2.3 |
| Age[h] | NS | | |
| Gender[i] | NS | | |
| CEA[f] | NS | | |
| TPA[f] | NS | | |

[a] Cox proportional hazards model. [b] Significance level. NS, non-significant. [c] Relative hazard. [d] Confidence interval at 95% level. [e] Dukes' stages entered as nominal groups and compared with Dukes' A stage. [f] The logarithms of the preoperative serum levels were entered as continuous variables. [g] Localisation as category (rectal, 0; colonic, 1). [h] Age was entered as a continuous variable. [i] Gender entered as category (male, 0; female, 1).

continuous variables in the Cox model, Dukes' stage was followed by CA 242 ($P<0.0001$), TPS ($P=0.0007$), hCGβ ($P=0.0017$) and tumour localisation ($P=0.0135$), whereas CEA, TPA, age and gender dropped out from the model as not being independent prognostic factors (Table IV).

### Discussion

This study confirmed that Dukes' stage is the strongest prognostic factor in colorectal cancer. However, when neoadjuvant treatment is used there is an increasing need to predict outcome already before surgery. For post-operative chemotherapy there is also a need to select patients with unfavourable prognosis, especially in Dukes' C cancer. Serum tumour markers could be useful for this purpose. Most other prognostic markers require tissue samples and are therefore available only after surgery (or biopsy) and the reproducibility of the results may be variable. In this aspect serum tumour markers differ from other prognostic tests, as the results are available preoperatively and they are reproducible and easily interpreted.

All the investigated tumour markers provided prognostic information. An elevated serum level of any of the investigated tumour markers was strongly related to disseminated disease and poor survival. CA 242 was the only marker that provided prognostic information within Dukes' B and C colorectal cancer. In Dukes' D colorectal cancer not only CA 242 but also TPA, TPS and hCGβ showed a prognostic value. In contrast to many previous reports, CEA did not provide significant prognostic information in addition to stage of disease (Wanebo *et al.*, 1978; Wolmark *et al.*, 1984; Brümmendorf *et al.*, 1985; Slentz *et al.*, 1994; Wang 1994; Lindmark *et al.*, 1995). In multivariate analysis CA 242, TPS and hCGβ were independent prognostic factors, when entering the serum tumour marker levels as continuous values. When entering the serum tumour marker levels as normal or elevated (dichotomised) in the model, hCGβ dropped out. In a recent report CA 242 was, as in our study, shown to be a prognostic factor (Lindmark *et al.*, 1995). However, in that report TPA and CEA were found to be superior to CA 242, and in that respect the results differed markedly from our results. In an earlier study by the same group, TPA was found to be a superior prognostic factor to CEA and CA 50 in rectal cancer (Ståhle *et al.*, 1988*b*).

Previously, CA 242 has been reported to be a valuable additional diagnostic tool preoperatively and in the follow-up of patients with colorectal cancer (Kuusela *et al.*, 1991; Nilsson *et al.*, 1992; Hall *et al.*, 1994; Carpelan-Holmström *et al.*, 1995). The results of this study further support previous findings, showing that the preoperative CA 242 serum level is an independent prognostic factor.

We conclude that the Dukes' stage is still the strongest prognostic factor in colorectal cancer and that CA 242 provides additional prognostic information within Dukes' stages B, C and D. TPS and hCGβ were also shown to be independent prognostic factors but the difference in survival was significant only in advanced disease. The promising results on the prognostic value of CA 242 needs to be evaluated in prospective studies on colorectal cancer.

**Acknowledgements**
The authors thank Wallac Oy, Beki Diagnostics AB and Sangtec Medical AB for kindly supplying the CA 242, TPS and TPA test kits respectively. This study has been supported by grants from Finska Läkaresällskapet, Stiftelsen Perkléns Minne and from Medicinska Understödsföreningen Liv och Hälsa.

### References

ALFTHAN H, HAGLUND C, ROBERTS P AND STENMAN UH. (1992). Elevation of free beta subunit of human choriogonadotropin and core beta fragment of human choriogonadotropin in the serum and urine of patients with malignant pancreatic and biliary disease. *Cancer Res.*, **52,** 4628–4633.

BOGENSCHÜTZ O, BRÜMMENDORF T, STAAB HJ, ANDERER FA AND KIENINGER G. (1986). Prognostic value of preoperative serum CEA level compared to clinical staging IV. Histological grading and tumor type in colorectal and gastric cancer. *J. Surg. Oncol.*, **32,** 165–173.

BRÜMMENDORF T, ANDERER FA, STAAB HJ, HORNUNG A, STUMPF E AND KIENINGER G. (1985). Prognostic value of preoperative serum CEA level compared to clinical staging: III. An Approach to scoring of prognostic factors in colorectal cancer. *J. Surg. Oncol.*, **28,** 263–269.

CARPELAN-HOLMSTRÖM M, HAGLUND C, KUUSELA P, JÄRVINEN H AND ROBERTS PJ. (1995). Preoperative serum levels of CEA and CA 242 in colorectal cancer. *Br. J. Cancer*, **71,** 868–872.

DE MELLO J, STRUTHERS L, TURNER R, COOPER EH, GILES GR AND THE YORKSHIRE REGIONAL GASTROINTESTINAL CANCER RESEARCH GROUP (1983). Multivariate analyses as aids to diagnosis and assessment of prognosis in gastrointestinal cancer. *Br. J. Cancer*, **48,** 341–348.

DEANS GT, PARKS TG, ROWLANDS BJ AND SPENCE RAJ. (1992). Prognostic factors in colorectal cancer. *Br. J. Surg.*, **79,** 608–613.

DHAR P, MOORE T, ZAMCHECK N AND KUPCHIK HZ. (1972). Carcinoembryonic antigen (CEA) in colonic cancer. *JAMA*, **221,** 31–35.

FILELLA X, MOLINA R, GRAU JJ, PIQUE M, GARCIA-VALDECASAS JC, ASTUDILLO E, BIETE A, BORDAS JM, NOVELL A, CAMPO E AND BALLESTA AM. (1992). Prognostic value of Ca 19.9 levels in colorectal cancer. *Ann. Surg.*, 55–59.

GOSLIN R, STEELE G, MACINTYRE J, MAYER R, SUGARBAKER P, GLEGHORN K, WILSON R AND ZAMCHECK N. (1980). The use of preoperative plasma CEA levels for the stratification of patients after curative resection of colorectal cancers. *Ann. Surg.*, **192,** 747–751.

HALL NR, STEPHENSON BM, PURVES DA AND COOPER EH. (1994). The role of CA-242 and CEA in surveillance following curative resection for colorectal cancer. *Br. J. Cancer*, **70,** 549–553.

HOHENBERGER P, SCHLAG PM, GERNETH T AND HERFARTH C. (1994). Pre- and postoperative carcinoembryonic antigen determinations in hepatic resection for colorectal metastases. *Ann. Surg.*, **219,** 135–143.

JASS JR, ATKIN WS, CUZICK J, BUSSEY HJR, MORSON BC, NORTHOVER JMA AND TODD IP. (1986). The grading of rectal cancer: historical perspectives and a multivariate analysis of 447 cases. *Histopathology*, **10,** 437–459.

KUUSELA P, HAGLUND C AND ROBERTS PJ. (1991). Comparison of a new tumour marker CA 242 with CA 19-9, CA 50 and carcinoembryonic antigen (CEA) in digestive tract diseases. *Br. J. Cancer*, **63**, 636–640.

LINDMARK G, BERGSTRÖM R, PÅHLMAN L AND GLIMELIUS B. (1995). The association of preoperative serum tumour markers with Dukes' stage and survival in colorectal cancer. *Br. J. Cancer*, **71**, 1090–1094.

MARCILLAC I, TROALEN F, BIDART JM, GHILLANI P, RIBRAG V, ESCUDIER B, MALASSAGNE B, DROZ JP, LHOMME C, ROUGIER P, DUVILLARD P, PRADE M, LUGAGNE P-M, RICHARD F, POYNARD T, BOHUON C, WANDS J AND BELLET D. (1992). Free human chorionic gonadotropin beta subunit in gonadal and nongonadal neoplasms. *Cancer Res.*, **52**, 3901–3907.

MOERTEL CG, O'FALLON JR, GO VL, O'CONNELL MJ AND THYNNE GS. (1986). The preoperative carcinoembryonic antigen test in the diagnosis, staging, and prognosis of colorectal cancer. *Cancer*, **58**, 603–610.

NILSSON O, JOHANSSON C, GLIMELIUS B, PERSSON B, NORGAARD-PEDERSEN B, ANDRÉN-SANDBERG Å AND LINDHOLM L. (1992). Sensitivity and specificity of CA 242 in gastrointestinal cancer. A comparison with CEA, CA50 and CA19-9. *Br. J. Cancer*, **65**, 215–221.

SLENTZ K, SENAGORE A, HIBBERT J, MAZIER WP AND TALBOTT TM. (1994). Can preoperative and postoperative CEA predict survival after colon cancer resection? *Am. Surg.*, **60**, 528–532.

STÅHLE E, GLIMELIUS B, BERGSTRÖM R AND PÅHLMAN L. (1988*a*). Preoperative serum markers in carcinoma of the rectum and rectosigmoid. I. Prediction of tumour stage. *Eur. J. Surg. Oncol.*, **14**, 277–286.

STÅHLE E, GLIMELIUS B, BERGSTRÖM R AND PÅHLMAN L. (1988*b*). Preoperative serum markers in carcinoma of the rectum and rectosigmoid. II. Prediction of prognosis. *Eur. J. Surg. Oncol.*, **14**, 287–296.

STEELE G, ELLENBERG S, RAMMING K, O'CONELL M, MOERTEL C, LESSNER H, BRUCKNER H, HORTON J, SCHEIN P, ZAMCHECK N, NOVAK J AND HOLYOKE ED. (1982). CEA monitoring among patients in multi-institutional adjuvant GI therapy protocols. *Ann. Surg.*, **196**, 162–169.

TURNBALL RB, KYLE K, WATSON FR AND SPRATT J. (1967). Cancer of the colon: the influence of the no-touch isolation technique on survival rates. *Ann. Surg.*, **166**, 420–427.

WANEBO HJ, RAO B, PINSKY CM, HOFFMAN RG, STEARNS M, SCHWARTZ MK AND OETTGEN HF. (1978). Preoperative carcinoembryonic antigen level as a prognostic indicator in colorectal cancer. *N. Engl. J. Med.*, **299**, 448–451.

WANG JY, TANG R AND CHIANG JM. (1994). Value of carcinoembryonic antigen in the management of colorectal cancer. *Dis. Colon Rectum*, **37**, 272–277.

WIGGERS T, ARENDS JW AND VOLOVICS A. (1988). Regression analysis in prognostic factors in colorectal cancer after curative resections. *Dis. Colon Rectum*, **31**, 33–41.

WOLMARK N, FISHER B, WIEAND HS AND HENRY RS. (1984). The prognostic significance of preoperative carcinoembryonic antigen levels in colorectal cancer. *Ann. Surg.*, **199**, 375–382.